# Paternal inheritance of growth in fish pursuing alternative reproductive tactics

**DOI:** 10.1002/ece3.570

**Published:** 2013-04-28

**Authors:** Sabine Wirtz-Ocaňa, Dolores Schütz, Gudrun Pachler, Michael Taborsky

**Affiliations:** Department of Behavioral Ecology, Institute of Ecology and Evolution, University of BernWohlenstr. 50a, 3032, Hinterkappelen, Switzerland

**Keywords:** Alternative life histories, cichlids, *Lamprologus callipterus*, paternal genetic effects, reproductive strategies

## Abstract

In species with indeterminate growth, age-related size variation of reproductive competitors within each sex is often high. This selects for divergence in reproductive tactics of same-sex competitors, particularly in males. Where alternative tactics are fixed for life, the causality of tactic choice is often unclear. In the African cichlid *Lamprologus callipterus*, large nest males collect and present empty snail shells to females that use these shells for egg deposition and brood care. Small dwarf males attempt to fertilize eggs by entering shells in which females are spawning. The bourgeois nest males exceed parasitic dwarf males in size by nearly two orders of magnitude, which is likely to result from greatly diverging growth patterns. Here, we ask whether growth patterns are heritable in this species, or whether and to which extent they are determined by environmental factors. Standardized breeding experiments using unrelated offspring and maternal half-sibs revealed highly divergent growth patterns of male young sired by nest or dwarf males, whereas the growth of female offspring of both male types did not differ. As expected, food had a significant modifying effect on growth, but neither the quantity of breeding substrate in the environment nor ambient temperature affected growth. None of the environmental factors tested influenced the choice of male life histories. We conclude that in *L. callipterus* growth rates of bourgeois and parasitic males are paternally inherited, and that male and female growth is phenotypically plastic to only a small degree.

## Introduction

Indeterminate growth generates age-related size variation of reproductive competitors, which may lead to enormous intrasexual size dimorphism (ISD; Taborsky 1999, 2008). A large ISD selects for divergence in optimal reproductive tactics of competitors particularly in males, with large individuals taking advantage of their ability to monopolize mate access (bourgeois tactic) and small ones parasitizing these males' reproductive effort (Taborsky 1994, 1997, 2008; Plaistow et al. 2004). This generates specialized alternative reproductive tactics (ARTs; Gross 1996; Oliveira et al. 2008). In general, parasitic males benefit from maturing early and thereby reducing mortality risk before reproduction, which compensates for their often moderate reproductive success. Frequency dependent selection can maintain an evolutionary equilibrium of tactic frequencies through selection on growth rate thresholds for parasitic male maturation (Maynard Smith 1982; Gross 1991a; Taborsky et al. 2008; Taborsky and Brockmann 2010). In contrast, species with determinate growth show a much smaller ISD; in 490 species of passeriform birds listed by (Dunning 1992), for example, the largest males of a species were on average only 19% (or 1.19 times) heavier than their smallest male conspecifics (median; Taborsky 1999). Apparently, no specialized parasitic reproductive tactics fixed for life occur in passerine birds, even if they occur in other avian taxa (Jukema and Piersma 2006).

In the vast majority of cases, the tactics are chosen in dependence of current conditions (i.e., conditional tactics; Dominey 1984; Taborsky 1994; Gross 1996), where individuals can adjust to a specific situation in dependence of their state when competing for reproduction. In some species, however, males performing ARTs do not switch between different tactics but instead choose a particular mating tactic for life. In the marine amphipod *Jassa marmorata*, for example, the determination of fixed male phenotypes with morphological and behavioral differences reflecting fighting and sneaking tactics is strongly influenced by diet quality (Kurdziel and Knowles 2002). In the horned beetle *Onthophagus taurus*, larval food quantity predictably determines the development of either horned or hornless males, irrespective of the paternal phenotype (Hunt and Simmons 1997; Moczek and Emlen 2000), and triggered by juvenile hormone or analogs (Emlen and Nijhout 1999, 2001).

**Figure 1:**
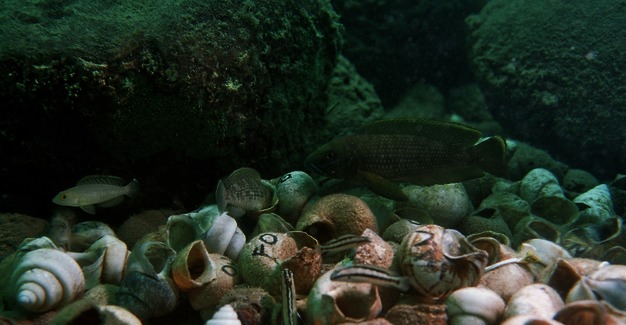
Field shot of a nest of *Lamprologus callipterus* consisting of empty snail shells. From right to left: nest male (dark), female (inspecting a shell), and dwarf male. In the foreground (middle), there are three specimens of a different species (*Telmatochromis vittatus*).

In the African cichlid *Lamprologus callipterus* (Fig. 1), males pursuing two highly specialized ARTs differ extremely in size, and these tactics are irreversible for life (Taborsky 2001). Large nest males collect, accumulate, and defend empty snail shells in which females lay eggs and care for the brood for 10 to 14 days (Sato 1994; Schütz and Taborsky 2000; Sato et al. 2004). Nest males must pass a size threshold to be able to carry shells and defend a breeding territory (Schütz and Taborsky 2000, 2005; Schütz et al. 2006). In contrast, dwarf males gain access to fertilizable eggs by entering a shell with a spawning female inside (“wriggling”, Sato et al. 2004) and hence have an upper size limit to fit in the shell. Specifically, dwarf males performing the wriggling tactic dive into a shell where a female is spawning, place their body alongside the female in the shell trying to wriggle past it in order to enter the inner whorl. If successful, dwarf males usually stay inside the shell until the end of spawning (Sato et al. 2004). Dwarf males mature early, halt growth long before reaching female size, and stay parasitic throughout life (Taborsky 2001). Apart from these two divergent spawning tactics that are linked to alternative male life histories, males of the bourgeois type may opportunistically try to fertilize eggs by releasing sperm in the shell opening when a female spawns with a nest male. This “sneaker tactic” is transitional and can be performed by males ranging from female to nest male size (see Schütz et al. 2006; Fig. 1 for a scheme of alternative life-history pathways in *L. callipterus*).

It is yet unclear how and when males decide to adopt the bourgeois or parasitic male life-history trajectories, and to what extent genetic and environmental factors are involved in this decision. This is of special interest in *L. callipterus*, because the extremely divergent size optima for males pursuing the nest male and dwarf male tactics in this species (cf. Sato et al. 2004) are likely to be associated with different life-history trade-offs of these male types, as body size strongly affects crucial fitness components through, for instance, its influence on predation risk, maturation, and fecundity. It has been shown that the optimal size and age at maturity can vary within species depending on environmental conditions (Roff 1984; Stearns 1992). Apart from the potential influence of genetic factors, environmental conditions that most likely influence growth and hence life-history decisions in *L. callipterus* include food abundance, temperature, and the availability of the potential breeding substrate, that is, empty snail shells, which are usually a limited resource (Sato and Gashagaza 1997). Ample food and high temperatures favor fast growth (Baum et al. 2005; Taborsky 2006), whereas at low temperatures metabolic rates are reduced, resulting in poor feeding and growth rates (Aune et al. 1997; Maclean and Metcalfe 2001; Baum et al. 2005). The number of available shells might influence the decision of males to adopt either the bourgeois or the dwarf male trajectory and hence could affect growth strategies. A high number of shells implies relatively low competition for this breeding substrate, so the nest male tactic should be more rewarding than if shells are very limited. In the latter case, it might be better to stay small and reproduce parasitically as a dwarf male.

Preliminary data from three broods raised in the laboratory (one each sired by a nest male, a sneaker male and a dwarf male, all of which were wild caught) suggested two different growth pathways in *L. callipterus* (Taborsky 2001). In this study, we investigated whether male and female growth patterns are heritable and to which extent they are determined by environmental factors. In a common garden experiment, we raised male and female offspring sired by nest or dwarf males to test for genetic effects on growth patterns in males and females, and on the determination of life-history pathways in males. For a part of the sample, maternal half-sibs were used to exclude maternal effects on growth parameters. We also tested for environmental influences on growth under standardized experimental conditions, by keeping juveniles with different combinations of food quantity, temperature, and abundance of snail shells in a full factorial design.

## Materials and Methods

### Genetic paternal effects on the growth of offspring

**Figure 2:**
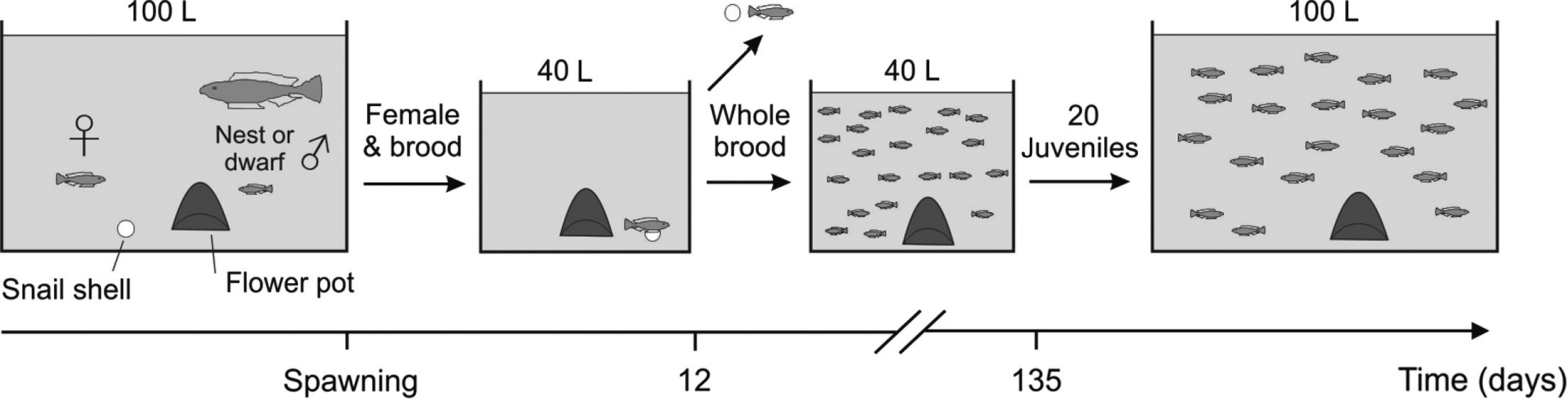
Experimental setup to test for genetic differences in growth patterns of males and females in dependence of male sire (*N* = 21 broods).

To test for potential genetic paternal effects on the determination of life-history pathways in males and females, we raised offspring of 21 broods sired by either a territorial or a dwarf male, respectively (experimental design see Fig. 2). All fish were fed ad libitum once a day with Tetra-Min Baby and TetraMin flakes for the first 4 months, then with TetraMin flake food and frozen food. The water temperature was kept constant at 25°C (±0.5°C). At the age of 4 months, 20 randomly chosen juveniles of each brood were transferred into experimental tanks (Fig. 2). From this point onwards, the young were measured once a month for >12 months (weight, standard, and total lengths). If <20 individuals had survived in a brood, the space for the rest of the brood was narrowed proportionally with help of an opaque partition in order to keep the density constant for all broods. Juvenile fish were sexed by checking the genital papillae; the last measurements per brood were taken between 1.97 and 2.42 years of age. The 21 broods used in this study were produced by 14 different females. Seven of these females produced one brood with two different males each (one nest male and one dwarf male, respectively), creating maternal half-sib pairs to control for maternal effects in our comparison of paternal genetic effects of the two male life histories (cf. Barber and Amott 2000).

The von Bertalanffy growth function (vBGF) describes the growth process as a differential equation of length (*L*) over time (*t*) (von Bertalanffy 1938). Growth (standard length, SL at age *t*) was modeled using the simplified vBGF (von Bertalanffy 1957): *L*_*t*_ = *L*_inf_ (1 – e ^−*K*(*t* - *t*^_0_^)^), with *L*_*t*_: size, here SL of the fish at time *t* (years); *L*_inf_: asymptote or maximum size achievable (length of a fish at infinite age); *K*: growth coefficient (rate which determines how fast the fish approaches its infinite length); and *t*_0_: a theoretical age at which size is theoretically zero (Bagenal 1978). From all broods raised in the laboratory, we calculated mean body lengths of male and female offspring sired by nest and dwarf males between 7 and 18 months of age. The vBGF was fitted separately for male and female offspring sired by either male type. As the fish could be sexed only from an age of 7 months onwards, data of unsexed fish were included in the model for the measurements obtained until 6 months of age. Note that in these analyses, sample sizes differ from *N*_terr_ = 10 and *N*_dwarf_ = 11 broods because in one of the territorial male broods only four growth measures could be obtained, which is why this brood was excluded when fitting the vBGF. In addition, two dwarf male broods did not contain females, one of which belonged to the maternal half-sib pairs.

### Environmental effects on the growth of offspring

**Figure 3:**
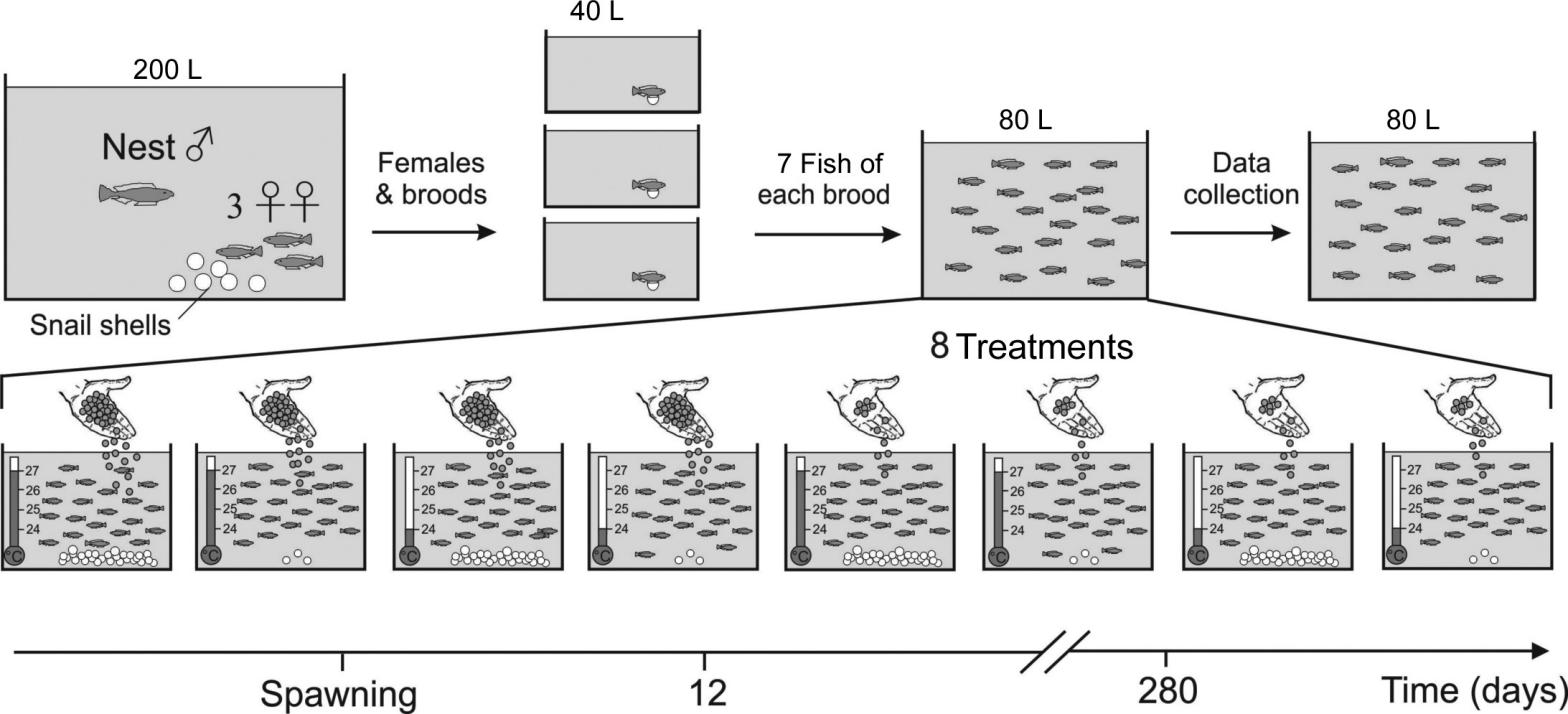
Experimental setup to test for environmental effects on growth rates in offspring of nest males. The number of shells in the tank (3 or 30), water temperature (24 or 27°C), and the amount of food (3 or 9% of body weight/day) were varied in a full factorial design. Tank sizes are depicted in liters (e.g., 100 L).

To experimentally test for the influence of environmental conditions on growth, three broods of one territorial male, which spawned with three different females (half-sib design), were raised in tanks with different combinations of food availability, temperature, and numbers of shells (for experimental design see Fig. 3). We used offspring of only one male in this experiment to exclude variance caused by genetic paternal effects on growth. To control for potential maternal effects on growth, offspring of the three females were distributed equally between the different setups, by putting seven fish of each brood into each of eight compartments, in total 21 fish per compartment. As in experiment 1, the tank size was adjusted using partitions to control for density effects due to different mortality between the tanks. Measuring the fish was only possible from a threshold size of 1.6 cm SL (age: 9 months), when handling did not involve a mortality risk. After all fish had reached this threshold-size, their SL, total length, and weight were determined every 4 weeks for a period of 6 months.

#### Food treatment

Before the first measurements, the food amount was either 2 mg of Mikromin for the low food treatment (determined as being sufficient for slow but steady growth in pilot trials) and three times this amount (6 mg) for the high food treatment. When the size measurements started, food was changed to Sera Vipan flake food, and the amount per day was adjusted to the total biomass of all fish (% of weight) in each tank. It was offered in high (9%) and low (3%) abundance, where 9% of the fish's biomass is equal to feeding them ad libitum and 3% allows to slow and steady growth (Taborsky 2006). Food was distributed over three feeding times per day. If fewer than 21 individuals had survived in a brood, the space for the rest of the brood was narrowed proportionally by an opaque partition in order to keep the density constant for all broods.

#### Shell treatment

Shells were offered in high (30) and low (3) numbers resembling high and low densities in the field (own observations).

#### Temperature treatment

Temperature was kept at either 24 or 27°C, which reflects the natural range at different depths and seasons in nature (own observations). Temperature was checked continuously by using automatic temperature loggers (“Hamster”) and maximum deviations were ±0.5°C. Due to mortality, the numbers of fish in each tank decreased considerably during the first 9 months and also during the measurement period. In one tank only one fish survived. The baseline mortality rate of juveniles in substrate breeding cichlids is generally high, which corresponds to the large clutch sizes they usually produce (mean = 95 eggs in *L. callipterus*; Schütz et al. 2012). The mortality rate of juveniles in our experiment did not seem to differ from baseline levels observed in standard holding conditions and in the field (S. Wirtz-Ocaňa, pers. obs.).

### Statistics

The distributions of data were tested for normality with the Kolmogorov–Smirnov normality test. Data that differed significantly from normal distributions (*P* < 0.1) were analyzed with nonparametric statistics. For all analyses, two-tailed tests were applied. To test for the genetic paternal influence on growth, we compared for all 21 broods whether the sizes of male/female offspring of nest males differ from those of dwarf males at the age of 12 months with *t*-tests. We used the Fisher's exact test for each brood to check for deviations from an expected 1:1 sex ratio. With Mann–Whitney *U*-tests, we checked for differences in the survival rate of offspring sired by dwarf males or nest males. For each sex, we tested for differences in the estimated growth rate, *K* and the infinite body size, *L*_inf_ between offspring sired by nest or dwarf males with *t*-tests. To check for differences in offspring body sizes sired by territorial and dwarf males from a very young age onwards, we used a univariate general linear model that compared body sizes (SL, dependent variable) of nest and dwarf male offspring up to an age of 0.9 years (males and females together) in dependence of their age (covariate), sire type (fixed factor), and the interaction between age and sire type, all nested for brood number. With Wilcoxon matched-pairs signed-ranks tests, we checked whether male and female offspring of maternal half-sib broods differ in the estimated growth rate, *K* and the infinite body size, *L*_inf_. To test for the influence of environmental factors on growth, data of the first 6 months of measurement were analyzed. We used a univariate general linear model (GLM) with SL at 6 months as a measure of growth as dependent variable and temperature, the amount of food and shells as fixed factors.

## Results

### Genetic paternal effects on the growth of offspring

**Figure 4:**
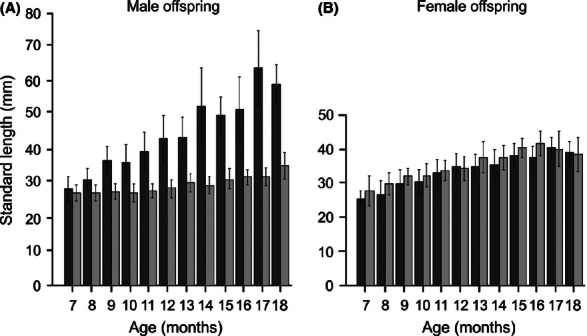
Mean standard lengths ±SD of (A) male and (B) female offspring sired by nest males (dark bars) or dwarf males (light bars) raised under standardized conditions. Male offspring: *N*_terr_ = 10 broods, *N*_dw_ = 11 broods, female offspring: *N*_terr_ = 10 broods, *N*_dw_ = 9 (two broods contained no females).

Male offspring of nest males and dwarf males showed highly divergent growth rates, whereas female offspring of both male types grew similarly quickly (Fig. 4). At an age of 12 months, male offspring of nest males had attained 43.34 ± 6.8 mm (SL, *N* = 38 fish from 7 broods) and male offspring of dwarf males 28.31 ± 2.54 mm (*N* = 29 fish from six broods, Fig. 4A), whereas female offspring sired by both male types had reached similar sizes (nest male sires: 34.7 ± 3.98 mm, *N* = 44 fish from six broods; dwarf male sires: 34.3 ± 3.54 mm, *N* = 50 females from six broods, Fig. 4B). This difference was significant for male offspring (*T*-test, *T* = 11.244, df = 65, *P* < 0.001), but there was no difference for female offspring (*T*-test, *T* = 0.573, df = 92, *P* = 0.568). In none of the broods, the sex ratio differed from 1:1 (Fisher's Exact Test, n.s. for all broods). We found no difference between the survival rates of offspring sired by nest and dwarf males (*U*-test: *Z* = −1.253, *P* = 0.218, *N* = 10 + 10 broods).

**Figure 5:**
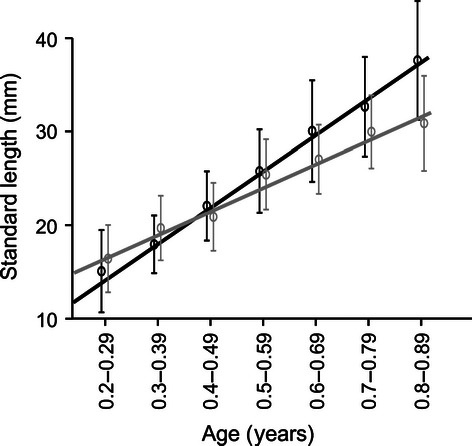
Body sizes of nest male and dwarf male offspring in different size classes up to an age of 0.9 years (arithmetic means, standard deviations, and regression lines) to show the switch point between early faster growth of dwarf male offspring and later faster growth of nest male offspring. Black bars and lines: nest male offspring (regression: *r* = 0.998, *P* < 0.001, *y* = 3.75x + 11.08), gray bars and lines: dwarf male offspring (regression: *r* = 0.99, *P* < 0.001, *y* = 2.50x + 14.53).

**Figure 6:**
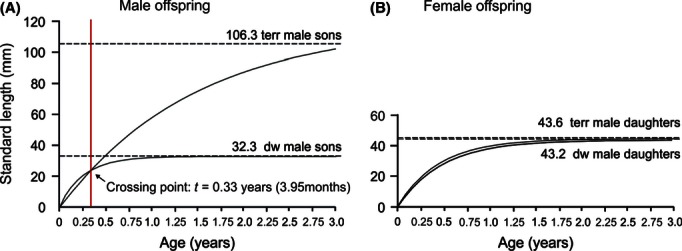
Calculated von Bertalanffy growth curves for (A) male and (B) female offspring sired either by nest males (terr) or dwarf males (dw). Dashed lines represent calculated maximal sizes (*L*_inf_). *L*_inf_ values are depicted in the graph, for values of *K* and *t*_0_ see Table 1.

Overall, the growth patterns of nest and dwarf male offspring differed significantly (GLM for the first 0.9 years, nested for brood, *r*^2^ = 0.613, df = 3, *F* = 526.33, *P* < 0.001). The age of fish (df = 1, *F* =1417.193, *P* < 0.001), as well as the sire type (df = 1, *F* = 32.915, *P* < 0.001) and the interaction between age and male type (df = 1, *F* = 67.943, *P* < 0.001) significantly determined the SL of offspring (Fig. 5). Male offspring of the two sire types differed significantly in the estimated growth rate, *K* and the infinite body size, *L*_inf_ (*P* < 0.001, Table 1A; vBGFs: see Appendix 1). The early growth rate estimate of male dwarf male offspring was more than five times higher than that of male nest male offspring, with dwarf males growing faster than nest males in the first 4 months of their lives (until SL = 23.77 mm, Fig. 6A). At this point the growth rates of male offspring crossed and henceforth nest male offspring grew faster than dwarf male offspring. The SL estimate at infinite age (*L*_inf_) for nest males exceeded that of dwarf males more than threefold (106.31 ± 43.3 mm vs. 32.31 ± 4.4 mm, *N* = 10 + 10 broods). We found no differences in any vBGF parameters between female offspring sired by nest male or dwarf male fathers (Table 1B and Fig. 6B). The von Bertalanffy growth parameters of the maternal half-sib broods are separately summarized in Appendix 2.

**Table 1:** Mean values for calculated parameters of the von Bertalanffy growth model for male (A) and female (B) offspring

	Sire	*N*	Mean	SD	*t*	df	*P*
(A)							
*K*	terr	10	0.77	0.29	−5.557	18	**<0.001**
	dw	10	4.04	1.84			
*L*_inf_	terr	10	106.31	43.30	5.375	18	**<0.001**
	dw	10	32.31	4.44			
*t*_0_	terr	10	0.107	0.046	0.309	18	0.761
	dw	10	0.089	0.178			
(B)							
*K*	terr	10	2.22	0.821	0.043	17	0.967
	dw	9	2.20	0.897			
*L*_inf_	terr	10	43.63	7.657	0.148	17	0.881
	dw	9	43.20	4.333			
*t*_0_	terr	10	0.198	0.241	1.183	17	0.253
	dw	9	0.092	0.1215			

Results of *t*-tests between offspring sired by nest (terr) or dwarf (dw) males. Arithmetic means and standard deviations (SD) are shown, significant differences are marked in bold.

### Environmental effects on the growth of offspring

**Figure 7:**
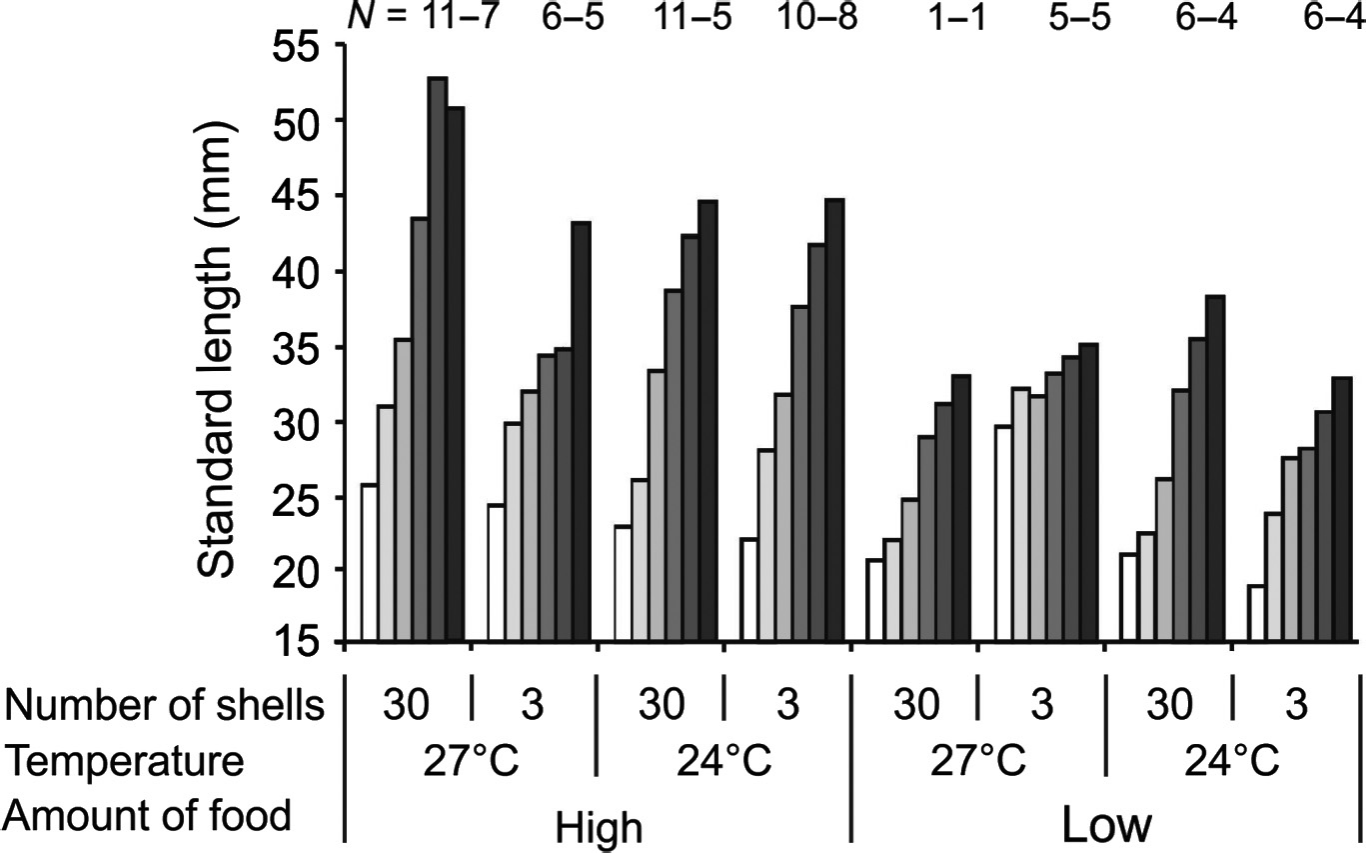
Mean standard length of a territorial male's offspring from three different females kept under different environmental conditions. *N*: Number of offspring at the beginning and end of measurement. Bars from white to black depict 1 month age classes, from 9 (white) to 14 (black) months of age.

The experimental variation of environmental conditions showed that dwarf males were not produced under any of the conditions. As predicted, food had a significant effect on growth (GLM, *F* = 5.318, df = 1, *P* = 0.03), but neither temperature (*F* = 0.826, df = 1, *P* = 0.372) nor the number of snail shells (*F* = 2.537, df = 1, *P* = 0.124) or any interactions between these parameters affected growth significantly (df = 1, 0.072 < F < 0.501; 0.124 < *P* < 0.877; Fig. 7).

## Discussion

Our data show that *L. callipterus* males pursuing ARTs fixed for life show divergent growth patterns that are genetically determined by the father. Environmental conditions do not determine the tactic choice of males and influence growth only to a minor degree. In contrast, the growth pattern of female offspring does not depend on the reproductive tactic of their father.

Von Bertalanffy growth models, obtained from our growth experiments examining genetic paternal effects on the determination of ARTs, gave a size estimate at infinite age (*L*_inf_) for dwarf males (SL = 32.3 ± 4.4 mm) that matches the average SL of 32.2 ± 4.7 mm of dwarf males employing the wriggling tactic in the field (Sato et al. 2004). As growth can be influenced by the degree of reproductive investment (Charnov 1993), dwarf males apparently invest all surplus energy in gonads, in turn reducing growth to nearly zero. In the field, the gonadosomatic index (GSI = ((gonad weight/1000)/body weight) ·100) of *L. callipterus* dwarf males (mean 1.99 ± 0.78 SD) exceeds that of nest males (0.37 ± 0.11) more than fivefold (Sato et al. 2004).

Dwarf males must be smaller than females to be able to successfully enter shells with spawning females inside, which explains their extremely small size (>40 times smaller than nest males; Taborsky 1999; Sato et al. 2004). Compared to male offspring of dwarf male sires, all female offspring and male offspring of nest males did not halt growth over the course of our measuring period. In our study population at Kasakalawe Bay, Lake Tanganyika, where nest males sometimes carry large shells over long distances, bourgeois males are also size restricted, but in the opposite direction than dwarf males and females (Schütz and Taborsky 2005). Experiments revealed that nest males need a minimum SL of 9 cm to be able to carry shells and defend their territories successfully (Schütz and Taborsky 2005). In the field, nest males and dwarf males show very different patterns of activity and energy allocation during reproduction (Schütz et al. 2010).

We found that dwarf males grow faster than nest males up to an age of ∼4 months, thereafter nest males grow faster. As offspring cannot be sexed and individually marked without a high mortality risk until an age of ∼8–9 months, we obtained a combined measure of male and female offspring of each brood until this age. As female offspring of the two male types grew at similar rates, the differences in growth rate estimates of male offspring of nest and dwarf males were reduced, that is, the real growth differences were very likely even greater.

The estimate of the initial growth rate of dwarf males was more than five times higher than that of nest males, whereas the SL estimate at infinite age for nest males exceeded that of dwarf males more than threefold. Growth estimates based on the von Bertalanffy growth model have been disputed (e.g., Lester et al. 2004), and it has been argued that this method should not be applied to model age and size at maturity (Day and Taylor 1997). However, application of the vBGF often proved to be successful, for example, providing the best growth model for 16 populations of fish (Chen et al. 1992) and for different goby species (Hernaman and Munday 2005). As we were not modeling age or size at maturity, we used the vBGF for describing growth in *L. callipterus* and the close match of our results with sizes of reproductively active individuals of different male types in the field suggests that this model describes the growth patterns in this species adequately.

As predicted, in our experiment varying environmental conditions to test for potential effects on the determination of ARTs, the growth of nest male offspring was influenced by the amount of food provided to them. The effect of food on growth and life-history traits such as fecundity and survival has been well documented (e.g., Nicieza and Metcalfe 1997; Segers and Taborsky 2011). For example, poor environmental conditions and low food availability early in life can result in reduced growth rates, smaller adult size, lower energy reserves, and inferior competitive ability, and therefore in reduced lifetime fitness (Taborsky 2006). Often, cold temperature can result in lower growth rates as well (e.g., Nicieza and Metcalfe 1997), but this effect did not emerge in *L. callipterus* within the range of natural temperatures. The correlation between temperature and growth rate is probably more pronounced in temperate regions than in the tropics (Aune et al. 1997; Nicieza and Metcalfe 1997), where the range of temperatures encountered by fishes is relatively small.

The number of snail shells in the environment also did not affect the growth rate of nest male offspring in our experiments. In the field, body size optima for the two sexes vary between populations, depending on the availability and sizes of empty shells. A comparative study on shell brooding cichlids in Lake Tanganyika revealed that nest males of *L. callipterus* collected in a shell-bed area in Burundi were on average much smaller (maximum SL = 51.1 mm) than males from a southern population in a rocky and sandy habitat (maximum SL = 115.9 mm), whereas females showed a similar size range (Gashagaza et al. 1995). In Burundi at the northern end of Lake Tanganyika, nest males and females have been observed to hide in *Neothauma tanganicense* shells when threatened by predators. In contrast, nest males in southern populations are far too big to enter shells and have never been found to hide in shells, but between rocks or in the sand (Sato 1994; Sato and Gashagaza 1997; Schütz and Taborsky 2000). It seems that nest male size of the Burundi-population is greatly influenced by natural selection due to high predation risk, causing hiding in shells to be essential. In an experiment where *L. callipterus* females of similar body size were housed under standardized conditions in aquaria equipped with either large *N. tanganicense* or small *Paramelania damoni* shells, females kept in tanks with large shells grew significantly heavier (*P* = 0.028), although no longer (*P* = 0.54), than females housed with small shells during a period of 18 months (Schütz and Taborsky 2005). This, together with our results, shows that growth and body size are phenotypically plastic in *L. callipterus*, but only to a small degree.

We should like to stress that we tested potential environmental effects only on nest male offspring, therefore we cannot exclude that growth patterns might be affected differently in young sired by dwarf males. Most importantly, however, environmental factors seem to have no influence on the choice of the male reproductive phenotype. As males of the nest male type can also pursue opportunistic sneaking behavior, environmental conditions nevertheless might influence the choice of this parasitic spawning behavior.

In *L. callipterus*, different male ARTs are fixed for life, and the diverging growth patterns of different male morphs are governed by a genetic polymorphism. This has not been documented yet from other fish species with male ARTs. In bluegill sunfish (*Lepomis macrochirus*), for example, depending on the population small parasitic males may be as old as large nesting males (Dominey 1980) or only half as old (Gross and Charnov 1980; Gross 1982), but like in *L. callipterus,* these reproductive tactics are fixed for life, at least from the point of sexual maturity onwards (c.f. Taborsky 1994). Back-calculations of growth from analysis of scales did not suggest different growth patterns of bourgeois and parasitic males in *L. macrochirus* in the field (Gross 1982), which has been confirmed by experimental evidence (Neff and Lister 2007). This and the divergent fitness estimates of different male types suggest that the alternative life histories in this species likely are not governed by a genetic polymorphism (Neff and Lister 2007). In several salmonid species, a genetic influence on precocious sexual maturation and the development of ARTs has been demonstrated. For example, in Chinook salmon (*Oncorhynchus tshawytscha*) where some males reach sexual maturity at least 1 year before other males (“jacking”), the father component of the additive genetic variance yielded a significantly higher heritability estimate than the mother component (Heath et al. 2002). However, in this species also rearing environment, particularly factors affecting growth, play a role in determining the incidence of precocious sexual maturation (Gross 1991b; Heath et al. 1994). It has been concluded therefore that jacking in chinook salmon is a polygenic trait that likely follows a threshold model, where the threshold is partially environmentally determined; or alternatively the threshold is fixed, but the phenotype depends on the environment (Heath et al. 2002). Also, male dimorphism in the corkwing wrasse *Symphodus melops*, with males either behaving territorial or as sneakers, has been reported to be fixed for life (Dipper 1981). However, in this species, the expression of male morph is likely dependent on the early growth pattern, as males adopting sneaking later in life are smaller after the first growth period than future territorial males (Uglem et al. 2000).

We conclude from our results that in *L. callipterus* growth rates of nest males and dwarf males are heritable in the male sex, which determines two alternative reproductive phenotypes that are fixed for life. Male and female body sizes are phenotypically plastic to only a little degree, and the different body sizes reported from different populations of this species are probably reflecting adaptations to divergent optima caused by local differences in predation pressure and breeding substrate. However, the genetic and environmental influence on body size differences between populations needs further experimental scrutiny.

## Appendix 1: von Bertalanffy growth parameters *L*_inf_, *K* and *t*_0_ and the correlation coefficient *r*^2^ of the goodness of fit for the curve estimation

**Table:**

#	sire	*L*_inf_	*K*	*t*_0_	*r*^2^	Age min	Age max	*N*
Both sexes combined								
1	terr	63.18	1.02	0.03	0.718	0.28	1.50	19-17
2	terr	58.35	1.04	0.02	0.797	0.34	1.36	6-6
3	terr	52.47	1.58	0.14	0.595	0.44	1.39	20-20
4	terr	57.19	1.62	0.16	0.727	0.36	1.32	19-13
5	terr	73.49	0.88	0.18	0.556	0.37	1.15	19-15
6	terr	46.82	1.30	0.08	0.806	0.34	0.97	20-18
7	terr	46.52	1.72	0.08	0.860	0.34	1.09	20-17
8	terr	45.64	1.83	0.15	0.769	0.33	1.02	20-16
9	terr	58.62	1.34	0.14	0.850	0.34	1.00	11-8
10	terr	83.57	0.86	0.07	0.903	0.34	0.98	9-5
11	dw	33.09	1.92	−0.29	0.586	0.25	1.55	4-1
12	dw	34.27	3.22	0.10	0.767	0.29	1.52	13-7
13	dw	40.07	2.18	0.08	0.696	0.29	1.35	20-17
14	dw	35.77	3.37	0.25	0.571	0.48	1.27	20-17
15	dw	35.89	3.63	0.21	0.451	0.47	1.87	17-15
16	dw	32.58	2.97	0.06	0.800	0.22	1.08	20-18
17	dw	35.86	2.41	0.02	0.720	0.26	1.07	20-19
18	dw	31.70	3.95	0.12	0.700	0.27	1.05	20-19
19	dw	33.48	3.58	0.19	0.676	0.38	1.25	20-17
20	dw	48.88	0.98	−0.10	0.667	0.23	0.97	5-4
Males								
1	terr	86.59	0.92	0.11	0.920	0.28	1.50	7
2	terr	191.57	0.29	0.04	0.920	0.34	1.36	1
3	terr	151.50	0.43	0.16	0.850	0.44	1.39	8
4	terr	93.60	0.83	0.15	0.930	0.36	1.94	6
5	terr	148.06	0.48	0.05	0.890	0.37	1.15	7
6	terr	66.94	0.91	0.11	0.944	0.38	1.25	7
7	terr	60.69	1.09	0.05	0.932	0.34	1.09	7
8	terr	74.08	1.08	0.16	0.899	0.33	1.02	5
9	terr	108.23	0.63	0.13	0.937	0.34	1.00	4
10	terr	81.79	1.02	0.11	0.940	0.34	0.98	3
11	dw	33.14	1.90	−0.30	0.590	0.25	1.55	2
12	dw	34.56	3.54	0.12	0.820	0.29	1.42	3
13	dw	31.60	3.28	0.09	0.770	0.29	1.35	6
14	dw	31.04	7.42	0.35	0.690	0.48	1.58	3
15	dw	32.13	4.17	0.19	0.500	0.47	1.87	7
16	dw	29.24	3.94	0.08	0.859	0.22	1.08	13
17	dw	30.12	3.87	0.07	0.741	0.26	1.07	11
18	dw	29.67	5.03	0.15	0.898	0.27	0.97	8
19	dw	27.90	6.09	0.23	0.822	0.34	0.97	6
20	dw	43.67	1.15	−0.09	0.588	0.23	1.05	1
Females								
1	terr	45.13	1.69	0.06	0.850	0.28	2.42	10
2	terr	47.00	1.42	0.05	0.890	0.34	1.36	4
3	terr	41.49	2.52	0.18	0.740	0.44	1.39	11
4	terr	46.71	2.22	0.17	0.870	0.36	1.94	6
5	terr	37.13	4.10	0.16	0.660	0.37	1.15	8
6	terr	38.33	1.94	0.12	0.953	0.34	0.97	10
7	terr	40.83	2.19	0.87	0.874	0.34	1.09	10
8	terr	38.68	2.17	0.13	0.911	0.33	1.02	11
9	terr	38.21	2.78	0.18	0.877	0.34	1.00	4
10	terr	62.81	1.16	0.06	0.921	0.34	0.98	2
11	dw	40.80	2.04	0.05	0.900	0.29	1.61	2
12	dw	50.71	1.50	0.05	0.890	0.29	1.35	10
13	dw	40.84	4.11	0.33	0.840	0.48	1.58	13
14	dw	49.75	1.91	0.17	0.750	0.47	1.25	6
15	dw	41.86	1.79	0.02	0.894	0.22	1.08	5
16	dw	43.39	1.75	0.01	0.845	0.26	1.07	8
17	dw	39.43	2.90	0.12	0.928	0.27	1.07	7
18	dw	38.37	2.67	0.17	0.852	0.38	1.25	11
19	dw	43.67	1.15	−0.09	0.767	0.23	0.97	3

Age min and Age max correspond to the age (years) when the brood was measured for the first and last time. *N* indicates the number of fish per brood at the start and end of the measurements, respectively, for pooled data (sex = 0), and the number of individuals at the end of the measurement for males (sex = 1) and females (sex = 2) separately.

## Appendix 2: Offspring of maternal half-sib pairs only

**Table:**

	Male offspring				Female offspring			
		
	terr sires		dw sires		terr sires		dw sires	
				
	*L*_inf_	*K*	*L*_inf_	*K*	*L*_inf_	*K*	*L*_inf_	*K*
1	86.59	0.92	33.14	1.90	45.13	1.69	No females	
2	191.57	0.29	34.56	3.54	47.00	1.42	40.80	2.04
3	148.06	0.48	32.13	4.17	37.13	4.10	49.75	1.91
4	60.69	1.09	29.24	3.94	40.83	2.19	41.86	1.79
5	66.94	0.91	27.90	6.09	38.33	1.94	38.37	2.67
6	81.79	1.02	30.12	3.87	62.81	1.16	43.39	1.75
7	74.08	1.08	43.67	1.15	38.68	2.17	43.67	1.15
Mean	101.39	0.83	32.97	3.52	44.27	2.1	42.98	1.89

Male offspring sired by nest males had larger *L*_inf_ and smaller *K*-values than male offspring from dwarf males (Wilcoxon test, *Z* = −2.366, *P* = 0.018 and *Z* = 2.371, *P* = 0.018, *N* = 7 + 7). There were no differences in the two parameters for female half-sibs (Wilcoxon test, *Z* = −0.105, *P* = 0.971 and *Z* = −0.314, *P* = 0.753, *N* = 6 + 6). The table shows the von Bertalanffy growth parameters *L*_inf_ and *K* in the maternal half-sib broods. Each pair corresponds to the offspring of one female paired with a nest male (terr sires) and dwarf male (dw sires) in two consecutive broods produced in random order.
